# Comment on “Subcutaneous fat area at the upper thigh level is a useful prognostic marker in the elderly with femur fracture” by Kim et al.

**DOI:** 10.1002/jcsm.13140

**Published:** 2022-12-15

**Authors:** Youn‐Jung Kim, Kyung Won Kim, Won Young Kim

**Affiliations:** ^1^ Department of Emergency Medicine University of Ulsan College of Medicine, Asan Medical Center Seoul South Korea; ^2^ Department of Radiology University of Ulsan College of Medicine, Asan Medical Center Seoul South Korea

Wang et al.^1^ raised valuable questions for the paper ‘Subcutaneous fat area at the upper thigh level is a useful prognostic marker in the elderly with femur fracture’.^2^ The authors included consecutive elderly patients (aged ≥65) who were diagnosed with a proximal femur fracture at the emergency department (ED) between January 2010 and December 2017. We agreed with Wang's concern that body tissue composition might be affected by a time interval after injury. However, the median hours from injury to pelvic bone computed tomography (CT) scan was 10.7 h [interquartile range (IQR), 6.2–26.8] and 83.9% of patients underwent CT scans within 48 h after injury. All study patients underwent pelvic bone CT at the ED before hospital admission or surgery. This homogenous cohort means our results might be less affected by body composition changes during bed rest or surgery. We measured both thighs because selecting one side could be another bias.

Regarding the effect of tube voltage on muscle density, we did not adjust the potential variations in the muscle density according to the kVp and mAs, based on the following reasons. Firstly, according to our recent paper, which evaluated the effects of various CT acquisition protocols and CT vendors using an abdominal muscle/fat phantom, the mean muscle density increased with low tube voltage of 80 kVp compared with 120 kVp (difference range, 3.4–3.5 HU).^3^ In that experiment, we observed that the mean muscle density difference in each 10 kVp gap was less than 1 HU, which might be too minimal to influence on main results of this study. Secondly, CT was scanned with 120 kVp in all patients; thus, the effects of tube voltage on the main results of this study would be very minimal.

Regarding Figure 3B, we admit that we selected a too representative case with scanty subcutaneous fat and abundant muscle mass. The median muscle density of survivor and non‐survivor groups were 18.7 HU (IQR, 12.4–25.9) and 19.8 HU (IQR, 11.2–27.5), respectively. The muscle density in Table 2 is expressed as mean and standard deviation.

Wang et al. reported that most muscle parameters, particularly gluteus maximus and gluteus medius/minimus muscle, were statistically significantly higher in surviving patients. This is contrasted by our results, subcutaneous fat area (SFA) was an independent predictor for 1‐year mortality (adjusted hazard ratio, 0.987; 95% CI, 0.982–0.992; *P* < 0.001) in patients with proximal femur fracture. The possible explanation is the difference in the study population. We included only elderly patients, whose median age is 79 years, and mostly female (73.7%). Among the elderly, muscle mass already decreased, which is the possible reason for no association between muscle parameters and outcome, especially in females. A positive association was observed between SFA and survival duration in patients with cancers and female patients with end‐stage liver disease, which support our assumption.^4^, ^5^, ^6^, ^7^ Different covariates in the analysis would be another reason. When age, sex, mobility function before the trauma and body composition including skeletal muscle area (SMA), subcutaneous fat area (SFA) and SMA attenuation were adjusted in multivariate Cox proportional hazard analysis, SFA [adjusted hazard ratio (HR), 0.985; 95% confidence interval (CI), 0.980–0.990; *P* < 0.001] and SMA attenuation (adjusted HR, 0.958; 95% CI, 0.939–0.978; *P* < 0.001) were significantly associated with 1‐year mortality. However, after adjusting all the other variables that were shown to significantly affect 1‐year mortality in the univariate Cox proportional hazards regression analyses, SFA was the only significant body composition parameter. Our results suggested that SFA would be a simple and reliable screening tool for poor outcomes in elderly patients with proximal femur fracture.

## Conflict of interest

The authors declare that they have no competing interests.

## Funding

This work was supported by grants from the National Research Foundation of Korea (NRF‐2018R1D1A1B07050136) and the Korea Health Technology R&D Project through the Korea Health Industry Development Institute (HI18C1216).

## References

[1] Wang L , Yin L , Yang M , Cheng X . Muscle composition and the imminent mortality risk after hip fracture. J Cachexia Sarcopenia Muscle 2022. 10.1002/jcsm.13090. Epub ahead of print. PMC9745496 36259275

[2] Kim YJ , Seo DW , Ko Y , Hong SI , Kim KW , Kim WY . Subcutaneous fat area at the upper thigh level is a useful prognostic marker in the elderly with femur fracture. J Cachexia Sarcopenia Muscle 2022;12:2238–2246. 10.1002/jcsm.12845 PMC8718083 34708563

[3] Kim DW , Ha J , Ko Y , Kim KW , Park T , Lee J , et al. Reliability of skeletal muscle area measurement on CT with different parameters: a phantom study. Korean J Radiol 2021;22:624–633. 33569929 10.3348/kjr.2020.0914 PMC8005347

[4] Ebadi M , Tandon P , Moctezuma‐Velazquez C , Ghosh S , Baracos VE , Mazurak VC , et al. Low subcutaneous adiposity associates with higher mortality in female patients with cirrhosis. J Hepatol 2018;69:608–616. 29709682 10.1016/j.jhep.2018.04.015

[5] Ebadi M , Martin L , Ghosh S , Field CJ , Lehner R , Baracos VE , et al. Subcutaneous adiposity is an independent predictor of mortality in cancer patients. Br J Cancer 2017;117:148–155. 28588319 10.1038/bjc.2017.149 PMC5520211

[6] Antoun S , Bayar A , Ileana E , Laplanche A , Fizazi K , di Palma M , et al. High subcutaneous adipose tissue predicts the prognosis in metastatic castration‐resistant prostate cancer patients in post chemotherapy setting. Eur J Cancer 2015;51:2570–2577. 26278649 10.1016/j.ejca.2015.07.042

[7] Takeoka Y , Sakatoku K , Miura A , Yamamura R , Araki T , Seura H , et al. Prognostic effect of low subcutaneous adipose tissue on survival outcome in patients with multiple myeloma. Clin Lymphoma Myeloma Leuk 2016;16:434–441. 27263047 10.1016/j.clml.2016.04.010

